# Ratanasampil alleviates cognitive impairment in mice induced by chronic hypoxia by modulating inflammatory responses

**DOI:** 10.3389/fimmu.2026.1872294

**Published:** 2026-07-06

**Authors:** Jie Meng, Hongxiu Liang, Xiaolei Wu, Qiang Liu, Aiqin Zhu

**Affiliations:** 1 Institute of Geriatric, Qinghai Provincial People's Hospital, Xining, China; 2 Department of Geriatrics and National Clinical Research Center for Geriatrics, West China Hospital, Sichuan University, Chengdu, Sichuan, China; 3 Graduate School of Qinghai University, Qinghai University, Xining, China; 4 Department of Cancer Center, Army Medical Center, Army Medical University, Chongqing, China; 5 Department of Neurology, The First Affiliated Hospital of Guangzhou Medical University, Guangzhou, China

**Keywords:** cognitive impairment, hypoxia, Inflammation, peripheral inflammation, Ratanasampil

## Abstract

**Background:**

Chronic hypoxia at high altitudes contributes to cognitive decline, but effective therapies are lacking. The traditional Tibetan medicine Ratanasampil (RNSP) has neuroprotective properties, yet its effects on hypoxia−induced cognitive impairment and associated inflammation remain unknown.

**Methods:**

Aged C57BL/6J mice were exposed to chronic hypobaric hypoxia with or without RNSP pretreatment (7 mg/kg/d, 7 days plus concomitant administration). Cognitive function was assessed by Morris water maze and open field tests. Neuroinflammation, amyloid−β (Aβ) pathology, peripheral immune cell profiles (spleen and blood), and serum cytokine levels were analyzed.

**Results:**

RNSP treatment significantly improved spatial learning and memory deficits and reduced anxiety−like behavior induced by chronic hypoxia. In the brain, RNSP lowered the expression of IL−1β, IL−6, and TNF−α in the hippocampus and cortex. In peripheral tissues, RNSP reversed hypoxia−induced reductions in splenic and circulating CD3^+^/CD4^+^ T cells, decreased CD11b^+^ myeloid cell accumulation in the spleen, and attenuated gut inflammation. Moreover, RNSP rebalanced the serum cytokine milieu by decreasing pro−inflammatory mediators (IL−1β, IL−6, TNF−α) while restoring anti−inflammatory cytokines (IL−4, IL−10, TGF−β).

**Conclusion:**

RNSP alleviates chronic hypoxia−induced cognitive impairment through dual anti−inflammatory actions on both the central nervous system and peripheral immune compartments. These findings support RNSP as a promising therapeutic candidate for high−altitude cognitive decline and possibly other inflammation−related neurological disorders.

## 1 Introduction

Cognitive decline in individuals living at high altitudes has become an increasingly recognized issue, with growing evidence suggesting that chronic exposure to hypoxia may contribute to neurocognitive impairment. High-altitude environments, typically characterized by reduced oxygen availability, can lead to hypoxic stress, which affects various physiological and neurological processes. Epidemiological studies have shown that the prevalence of cognitive impairment and dementia among older adults living at high altitudes is significantly elevated. A systematic review and meta−analysis reported overall prevalence rates of 22.0% for cognitive impairment and 11.0% for dementia in high−altitude populations, approximately twice that of low−altitude regions (1). A comparative study in China found that older adults living at high altitude had a 2.92−fold higher risk of cognitive impairment compared to those at low altitude (2). Among Tibetan hypertensive patients, high−altitude residence was associated with a 2.38−fold increased risk of cognitive dysfunction (3). Studies indicate that long-term exposure to hypoxia can impair memory, learning, and other cognitive functions, especially in elderly individuals (4, 5). The pathophysiology of this cognitive decline is thought to involve a combination of chronic hypoxia-induced neuroinflammation, oxidative stress, and altered cerebral blood flow, leading to neuronal damage and dysfunction (6). In regions such as Tibet, where populations have lived at high altitudes for centuries, studies have shown a mixed impact of chronic hypoxia on cognitive function. While Tibetan populations have developed genetic and physiological adaptations that allow them to cope with low oxygen levels, some studies suggest that prolonged exposure to hypoxia may still lead to subtle cognitive impairments, particularly in elderly individuals (7, 8). Additionally, the accumulation of amyloid-beta plaques, a hallmark of Alzheimer’s disease, has been observed in hypoxic conditions, further indicating that high-altitude living may exacerbate neurodegenerative processes (9). Despite these findings, there is a significant gap in therapeutic approaches aimed at mitigating hypoxia-induced cognitive deficits. Understanding the mechanisms underlying these effects and identifying potential interventions are critical for addressing cognitive impairment in high-altitude populations.

Cognitive decline, particularly in age-related neurodegenerative diseases such as Alzheimer’s disease, is closely linked to both central and peripheral inflammation. In the central nervous system (CNS), neuroinflammation is a hallmark feature of cognitive decline and neurodegeneration. Microglia, the resident immune cells of the brain, become activated in response to injury, infection, or environmental stressors, leading to the release of pro-inflammatory cytokines, chemokines, and reactive oxygen species (ROS). Chronic hypoxia has been shown to critically involve microglia-mediated neuroinflammation. Microglial activation and subsequent myelin damage contribute to white matter injury and cognitive deficits in hypoxic conditions (10). Chronic intermittent hypoxia induces hippocampal microglial activation and excessive synaptic phagocytosis via astrocyte-microglia crosstalk, leading to learning and memory impairments (11). Hypoxia also increases reactive oxygen species and pro-inflammatory molecules, which can directly or indirectly activate microglia through vagal afferent pathways (12). Furthermore, hypoxia exerts profound molecular effects on neurons, particularly in the hippocampal CA1 region, where delayed and selective neuronal damage occurs (13). Chronic activation of microglia and astrocytes in the brain can contribute to synaptic dysfunction, neuronal damage, and the accumulation of amyloid-beta plaques, all of which are implicated in the pathogenesis of Alzheimer’s disease (14, 15). In parallel, peripheral inflammation has been shown to influence brain function. Systemic inflammation, often driven by chronic conditions such as obesity, diabetes, or cardiovascular disease, can lead to the activation of immune cells in peripheral tissues like the spleen, gut, and adipose tissue. These cells, including T cells and macrophages, release inflammatory mediators that can cross the blood-brain barrier and exacerbate neuroinflammation, further contributing to cognitive decline (16, 17). The gut-brain axis has emerged as a critical pathway linking peripheral immune activation to CNS inflammation. Gut dysbiosis induced by hypoxia can increase intestinal permeability, allowing bacterial products and cytokines to enter the circulation and amplify systemic inflammation (18). Alterations in gut microbiota composition can trigger neuroinflammatory pathways that impact systemic immunity and disease susceptibility (19). Dysbiosis-induced gut barrier dysfunction and increased release of inflammatory mediators are key contributors to neuroinflammation in neurodegenerative diseases (20). Thus, the interplay between central and peripheral inflammation plays a critical role in the development and progression of cognitive impairment, and targeting inflammatory pathways in both compartments may offer therapeutic potential for preventing or slowing cognitive decline.

Ratanasampil (RNSP) is a traditional Tibetan medicine widely used for treating cognitive impairment and neurological disorders. It is a complex herbal formulation known for its anti-inflammatory, antioxidant, and neuroprotective properties. Previous studies have demonstrated its efficacy in alleviating neurodegeneration (21, 22). We have previously showed that RNSP exerts protective effects in human neuronal SH-SY5Y cells, a widely used *in vitro* model for studying neurodegenerative diseases (23). We found that RNSP treatment significantly reduced oxidative stress and enhanced cell survival under conditions of neurotoxic insult. Mechanistically, it was observed to modulate key signaling pathways involved in cellular stress responses, thereby reducing apoptosis and promoting neuronal resilience (23). However, further studies are needed to fully elucidate its mechanisms of action and efficacy *in vivo*.

Despite its well-documented neuroprotective properties, the effects of RNSP on cognitive decline and inflammation induced by chronic hypoxia remain unclear. In this study, we hypothesized that RNSP alleviates hypoxia-induced cognitive impairment by suppressing both central neuroinflammation and peripheral inflammatory responses. We investigate whether RNSP can mitigate hypoxia-induced neuroinflammation and peripheral inflammatory responses in exacerbating cognitive impairment. To test this hypothesis, we used a mouse model of chronic hypobaric hypoxia and evaluated cognitive function, neuroinflammation, amyloid-β pathology, immune cell profiles in the spleen and peripheral blood, as well as cytokine levels in the gut and serum. Our results provide the first *in vivo* evidence that RNSP effectively mitigates hypoxia-induced cognitive deficits by modulating inflammatory pathways in both the CNS and peripheral tissues. Understanding the potential of RNSP to address both central and peripheral inflammation under hypoxic conditions could provide valuable insights into its therapeutic applications for cognitive decline in high-altitude populations and related neurological disorders.

## 2 Materials and methods

### 2.1 Reagents

Ratanasampil (RNSP, Zhunzi Z63020062) was purchased from Qinghai Jinke Tibetan Medicine Pharmaceutical Co. Ltd. (Xining, China). Antibodies of rat anti-CD11b (557397), rat anti-F4/80 (566787), rat anti-CD4 (558107), rat anti-CD8a (553030), rat anti-CD19 (557655), and hamster anti-CD3e were purchased from biosciences pharmingen (BD, USA). QuantiTect Reverse Transcription Kit and Rotor-Gene SYBR Green RT-PCR Kit were purchased from Qiagen (Hilden, Germany).

### 2.2 Animals

C57BL/6J mice (12 months-old) were purchased from the Beijing Vital River Lab Animal Technology Co., and all animal experiments were approved and carried out according to the Animal Ethics Committee of Qinghai University. All mice were housed in a room with automatically controlled temperature (21–25 °C), relative humidity (45–65%), and light-dark (12–12h) cycles. The mice in each cage were divided into the following treatment groups for each of the experiments: a 28-day control group, a 28-day hypoxia group, and a treatment group (7 days of RNSP pretreatment (7mg/kg/d) + 28 days of hypoxia combined with RNSP administration). All mice underwent an acclimatization period of 7 days upon arrival in Xining. After 7 days, the control group was placed in their normal environment at an altitude of 2260 meters in Xining, Qinghai Province, and administered CMC-Na solution by gavage daily for 28 days. The hypoxia group was placed in a hypobaric chamber simulating an altitude of 5000 meters and administered CMC-Na solution by gavage daily for 28 days. The treatment group received RNSP pretreatment for 7 days, followed by 28 days of administration in a hypobaric chamber. Outside the chamber, the animals were kept in a clean, well-ventilated environment with a room temperature of 22 ± 2°C, natural lighting, free access to food and water, and their bedding was changed regularly each day. Each group consisted of ten male mice. For serum collection and tissue harvest, mice were deeply anesthetized by intraperitoneal injection of pentobarbital sodium (50 mg/kg). Blood was collected via cardiac puncture and allowed to clot for serum separation. For immunohistochemistry, mice were then transcardially perfused with ice-cold phosphate-buffered saline (PBS) followed by 4% paraformaldehyde (PFA) under deep anesthesia. After perfusion, mice were euthanized by an overdose of pentobarbital sodium (≥150 mg/kg, i.p.) followed by cervical dislocation. For molecular biology assays (qPCR) and flow cytometry, mice were not perfused; instead, after blood collection, they were euthanized directly by an overdose of pentobarbital sodium (≥150 mg/kg, i.p.) and cervical dislocation. Brain, spleen, and intestine tissues were then rapidly dissected. All efforts were made to minimize animal suffering. An application for ethical approval was submitted on 24 February 2022 and ethical approval was granted on the following day (No. 2022-91).

### 2.3 Morris water maze

The Morris Water Maze assesses mouse learning and memory through navigation and spatial probe trials. After acclimatization, visually competent mice undergo hidden platform training over five days, with escape latency recorded across four daily trials. In the final probe trial, the platform is removed, and time spent in the target quadrant and platform crossings are measured to evaluate memory retention. The water temperature is maintained at 18–22 °C, and the pool is surrounded by distinct visual cues.

### 2.4 Open field

The open field test is conducted in a behavioral test chamber measuring 50cm × 50cm × 30cm. The bottom is divided into 5×5 square grids, with the central area being a 30cm × 30cm area on the bottom of the chamber, and the remaining areas being the outer perimeter. A camera is fixed in the center of the top of the test chamber and connected to a computer screen. An opaque cylinder large enough to hold a mouse is suspended from the central square on the bottom of the test chamber. The experimenter quickly and gently places the mouse into the cylinder, and pulls on the other end of the rope hanging from the cylinder outside the mouse’s line of sight, allowing the mouse to move freely. Another experimenter, standing in front of the computer outside the mouse’s line of sight, simultaneously pulls on the rope and records the mouse’s behavior, including the time spent in the central area, the number of times it stands up, and the frequency of urination and defecation within 5 minutes. The entire experiment is conducted in a quiet environment at a normal temperature. After each experiment, the urine and feces of each animal were cleaned up, and the box was wiped with ethanol. The next mouse experiment was conducted after the odor had dissipated.

### 2.5 Real-time quantitative PCR (RT-PCR) analysis

Total RNA was extracted using RNAiso Plus according to the manufacturer’s instructions. 1 μg of total RNA was used for cDNA synthesis using the QuantiTect Reverse Transcription Kit (Qiagen, Hilden, Germany). After an initial amplification with a denaturation step at 95 °C for 5 m, followed by 30–40 cycles of denaturation at 95 °C for 5 s, annealing at 60 °C for 10 s, and extension at 72 °C for 30 s, a final extension at 72 °C for 5 m was done upon completion of the cycling steps. The cDNA was amplified in duplicate using a Rotor-Gene SYBR Green RT-PCR Kit (Qiagen) with a Corbett Rotor-Gene RG-3000A Real-Time PCR System. The data were evaluated using the RG-3000A software program (version Rotor-Gene 6.1.93, Corbett, Sydney, Australia). The sequences of primer pairs were described as follows: IL-1β: 5′-CAACCAACAAGTGATATTCTCCATG-3′ and 5′-GATCCACACTCTCAGCTGCA-3′; TNF-α: 5′-ATGGCCTCCCTCTCAGTTC-3′ and 5′-TTGGTGGTTTGCTACGACGTG-3′; IL-6: 5′-CTCCCAACAGACCTGTCTATAC-3′ and 5′-CCATTGCACAACTCTTTTCTCA- 3′; ROS: 5′-TAACATGAGCACTGTTCGGATT-3′ and 5′-GTACGTGTCCTCAATACCCTTG-3′; APP: 5′-TGAATGTGCAGAATGGAAAGTG-3′ and 5′-AACTAGGCAACGGTAAGGAATC-3′; β-actin: 5′-CTACCTCATGAAGATCCTGACC-3′ and 5′-CACAGCTTCTCTTTGATGTCAC-3′.

For data normalization, an endogenous control (actin) was assessed to control for the cDNA input, and the relative units were calculated by a comparative Ct method. All qRT-PCR experiments were repeated three times, and the results are presented as the means of the ratios ± SEM.

### 2.6 ELISA assay for Aβ, IL-1β, IL-6, TNF-α, IL-4, IL-10, IL-17, TGF-β

Serum levels of amyloid-beta (Aβ) and cytokines (IL-1β, IL-6, TNF-α, IL-4, IL-10, IL-17, TGF-β) were quantified using commercial enzyme-linked immunosorbent assay (ELISA) kits according to the manufacturers’ instructions. Briefly, blood samples were collected from mice and allowed to clot at room temperature for 30 minutes before centrifugation at 3000 × g for 15 minutes to obtain serum. The serum aliquots were stored at -80 °C until analysis. All samples were diluted appropriately to fall within the dynamic range of the respective standard curves. Standards and samples were added to the antibody-precoated wells and incubated. After a series of washes to remove unbound substances, a biotinylated detection antibody specific to each target was added, followed by incubation with a streptavidin-horseradish peroxidase (HRP) conjugate. The colorimetric reaction was developed using a tetramethylbenzidine (TMB) substrate, and the reaction was terminated with a stop solution. The optical density of each well was immediately measured at 450 nm using a microplate reader, and the concentration of each analyte was calculated by interpolating from the corresponding standard curve.

### 2.7 Immunohistochemical staining

Brain, spleen, and intestine tissues were collected and fixed in 4% paraformaldehyde for 24 hours at 4 °C, followed by paraffin embedding. Sections were cut to a thickness of 4-5 μm. For immunohistochemical analysis, the sections were deparaffinized in xylene and rehydrated through a graded ethanol series. Antigen retrieval was performed by heating the sections in sodium citrate buffer (10 mM, pH 6.0). Subsequently, endogenous peroxidase activity was quenched by incubation with 3% hydrogen peroxide for 15 minutes at room temperature. After blocking with 5% normal goat serum for 1 hour, the sections were incubated overnight at 4 °C with primary antibodies against Aβ, IL-1β, IL-6, and TNF-α. Following washes with PBS, the sections were incubated with a horseradish peroxidase (HRP)-conjugated secondary antibody for 1 hour at room temperature. The immunoreaction was visualized using a 3,3’-diaminobenzidine (DAB) substrate kit, and the sections were counterstained with hematoxylin. Finally, the sections were dehydrated, cleared, and mounted. Stained images were captured using a light microscope, and the expression levels of the target proteins were analyzed based on the intensity of brown DAB staining.

### 2.8 Flow cytometry

Single-cell suspensions were prepared from spleen tissues or peripheral blood. Spleens were mechanically dissociated and passed through a 70-μm cell strainer. Red blood cells (RBCs) were lysed using ACK lysis buffer. For peripheral blood, whole blood was collected in heparin-coated tubes, and RBCs were similarly lysed. The obtained cells were washed and resuspended in cold PBS containing 2% fetal bovine serum (FBS). For cell surface marker staining, approximately 1×10^6 cells per sample were incubated with fluorescently conjugated antibodies against CD3^+^, CD4^+^, and CD11b^+^, or corresponding isotype controls, for 30 minutes at 4 °C in the dark. After staining, cells were washed twice to remove unbound antibodies and resuspended in staining buffer.

For intracellular cytokine staining, cells were first stimulated with a cell activation cocktail (containing PMA and ionomycin) in the presence of a protein transport inhibitor for 4–6 hours. After stimulation, cells were stained for surface markers as described above, followed by fixation and permeabilization using a commercial intracellular staining kit according to the manufacturer’s instructions. The cells were then stained with antibodies against cytokines of interest. Data acquisition was performed immediately on a flow cytometer, and the results were analyzed using specialized software. Lymphocytes and myeloid cells were gated based on forward and side scatter properties, and the frequencies of specific immune cell populations were determined within the live cell gate.

### 2.9 Statistical analyses

The data are represented as the means ± standard error of the mean. The statistical analyses were performed by one-way ANOVA followed by *post-hoc* tests, using the GraphPad Prism software package (GraphPad Software, California, USA). A value of p < 0.05 was considered to indicate statistical significance.

## 3 Results

### 3.1 RNSP ameliorates chronic hypoxia-induced cognitive deficits in the Morris water maze and open field tests

**Figure 1:**
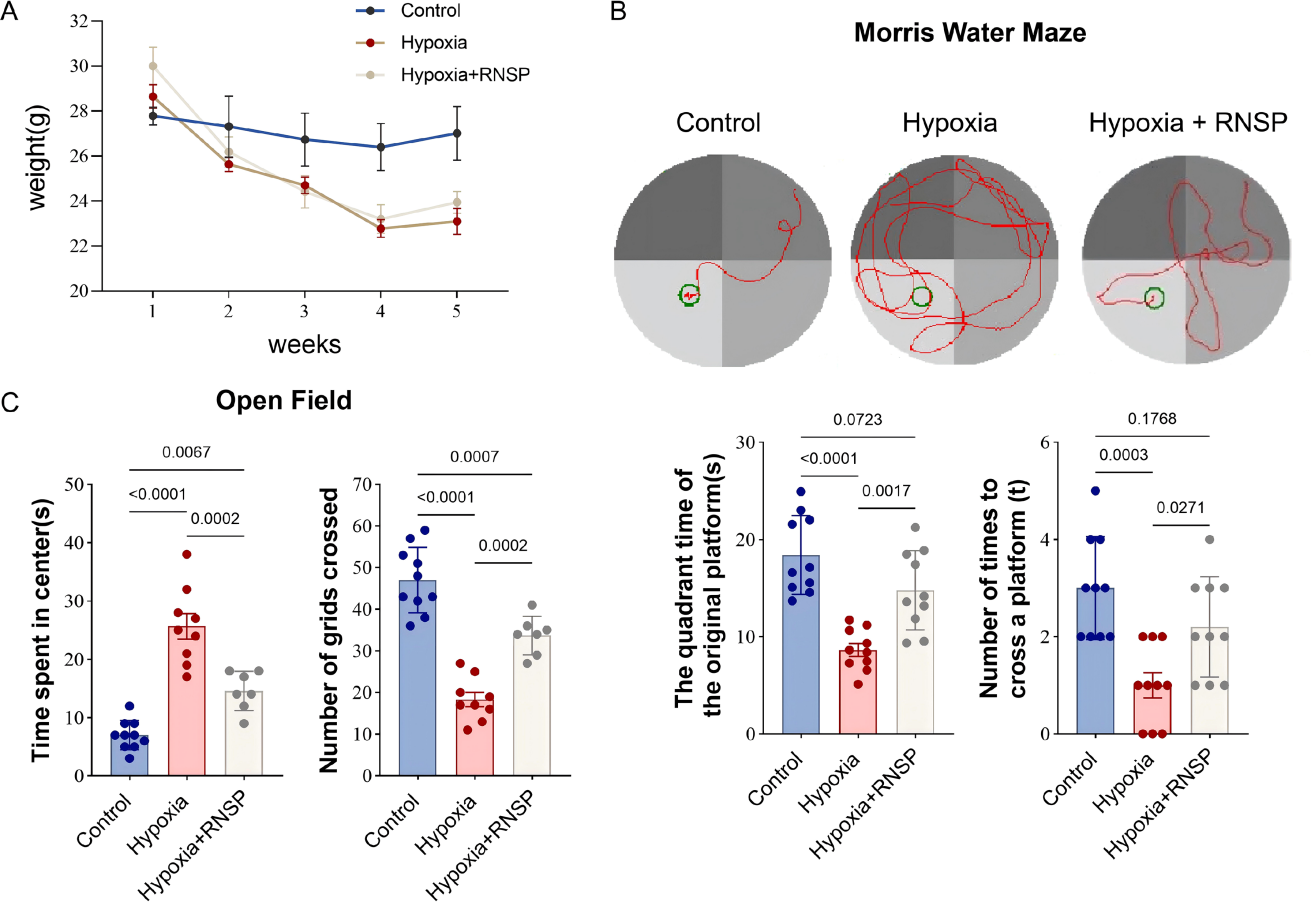
RNSP improved the chronic hypoxia-declined cognitive functions. **(A)** Body weight changes in control, hypoxia, and hypoxia + RNSP groups from week 1 to week 5. While the control group maintained a stable body weight, mice in the hypoxia group exhibited a progressive decline. RNSP-treated mice also showed an initial decrease in body weight, but demonstrated significant recovery by week 5. **(B)** Spatial memory performance assessed by the Morris water maze test. (Left) Time spent in the target quadrant during the probe trial. Hypoxia-exposed mice spent significantly less time in the target quadrant compared to controls, while RNSP treatment significantly restored the exploration time. (Right) Number of platform crossings during the probe trial. The hypoxia group showed a significant reduction in platform crossings, which was reversed by RNSP treatment. **(C)** Anxiety-like behavior evaluated in the open field test. (Left) Time spent in the center zone. Mice in the hypoxia group spent significantly less time in the center, indicating increased anxiety-like behavior, which was significantly improved by RNSP treatment. (Right) Total number of grid crossings in the open field. Hypoxia significantly reduced locomotor activity, an effect that was rescued following RNSP administration. Data are presented as mean ± SD (n = 10 per group). Statistical significance was determined by one-way ANOVA followed by *post-hoc* tests, with p < 0.05 considered significant. Exact p-values are shown directly above the bars.

To evaluate the protective effect of RNSP against chronic hypoxia-induced cognitive impairment, the mice were subjected to a 28-day chronic hypoxia protocol with or without RNSP pretreatment (7 mg/kg/d for 7 days) and concurrent administration. Throughout the 28-day period, the hypoxic and RNSP-treated hypoxic groups showed a comparable and steady decrease in body weight, but the body weight of the RNSP treatment group increased significantly in the later stage (**Figure 1A**). In the Morris water maze test, mice exposed to chronic hypoxia exhibited significant learning and memory deficits, manifested as a markedly longer escape latency during the training days and reduced time spent in the target quadrant during the probe trial compared to the normoxic controls. In contrast, RNSP-treated mice displayed significantly shorter escape latencies and spent more time in the target quadrant, demonstrating improved spatial learning and memory retention (**Figure 1B**). Furthermore, in the open field test, hypoxic mice showed increased anxiety-like behavior, characterized by a significant reduction in the time spent and distance traveled in the center area. RNSP treatment effectively mitigated this behavior, resulting in central area exploration times and activity levels comparable to those of the normoxic control group (**Figure 1C**). Collectively, these behavioral results confirm that RNSP pretreatment and concurrent administration significantly ameliorate the cognitive deficits and anxiety-like behaviors induced by chronic hypoxia in C57BL/6J mice.

### 3.2 RNSP attenuates neuroinflammation and reduces amyloid-beta accumulation in the hippocampus and cortex

**Figure 2:**
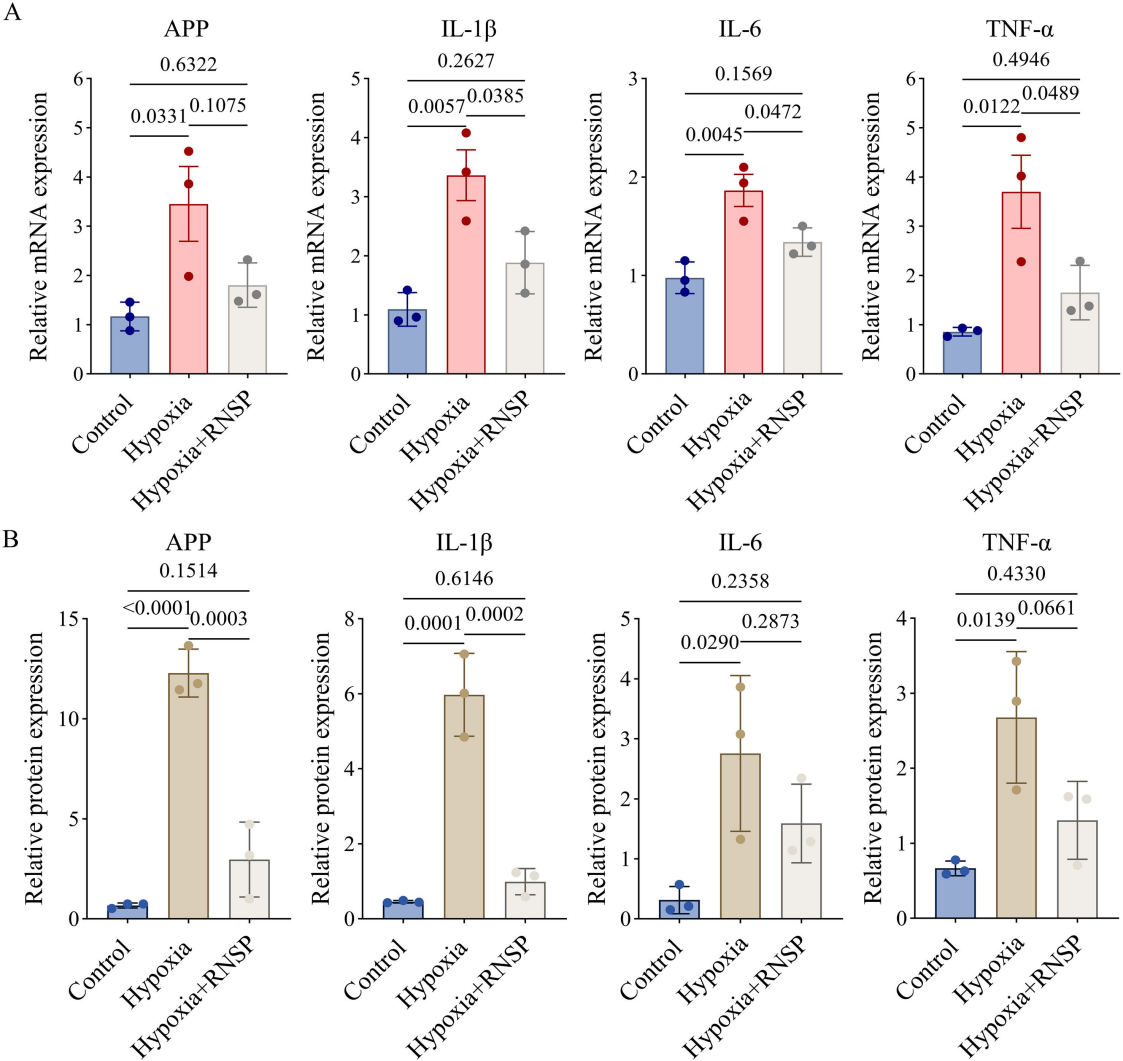
RNSP suppressed the chronic hypoxia-increased neuroinflammation in cortex and hippocampus. **(A)** mRNA expression levels of APP, IL-1β, IL-6, and TNF-α in the mouse cortex. **(B)** mRNA expression levels of APP, IL-1β, IL-6, and TNF-α in the mouse hippocampus. Data are presented as mean ± SD. Statistical significance was determined by one-way ANOVA followed by an appropriate *post-hoc* test, with p < 0.05 considered significant. Exact p-values are shown directly above the bars.

**Figure 3:**
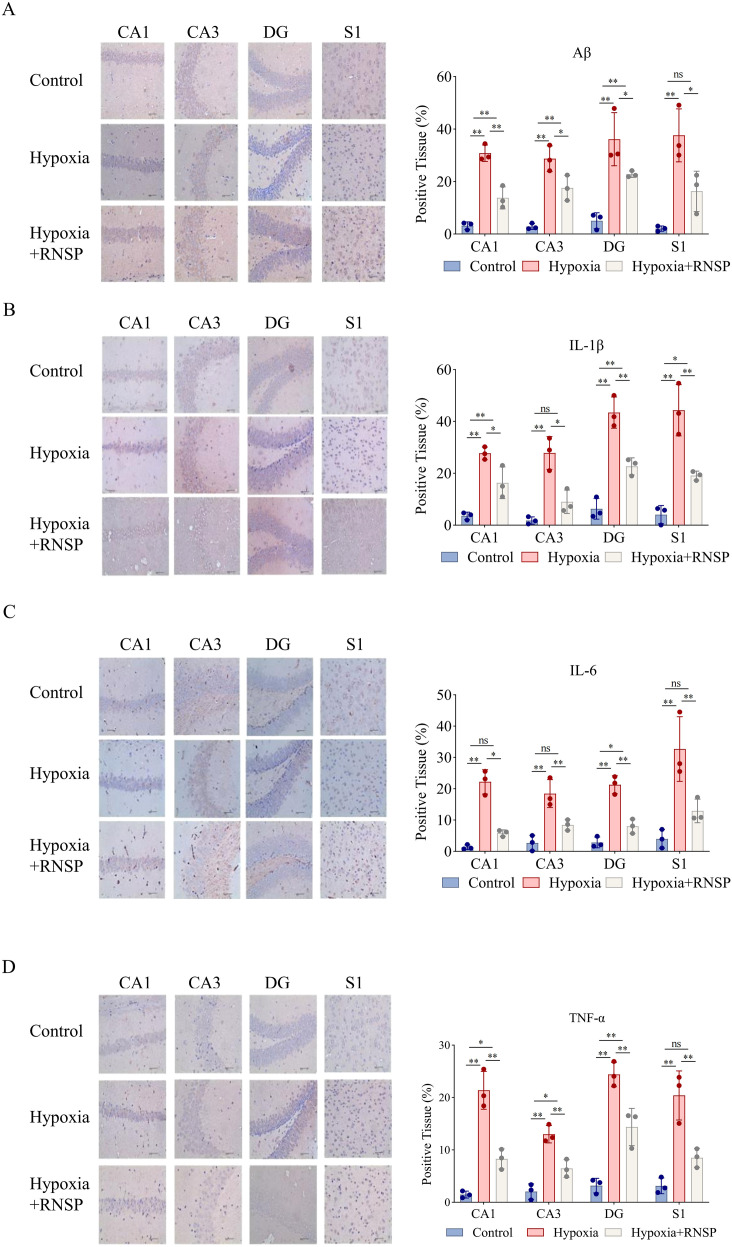
RNSP suppressed the chronic hypoxia-increased neuroinflammation in cortex and hippocampus. **(A–D)** The expression levels of inflammatory factors in the hippocampus of mouse brains were significantly increased by immunohistochemistry compared with the control group. The expression levels of Aβ, IL-1β, IL-6, and TNF-α in the CA1, CA3, DG, and S1 regions of the brain of mice in the hypoxia group were significantly increased. After 28 days of RNSP treatment, the expression of Aβ, IL-1β, IL-6, and TNF-α decreased. Data are presented as mean ± SD. Statistical significance was determined by one-way ANOVA followed by an appropriate *post-hoc* test. *p < 0.05, **p < 0.01 between the indicated groups.

To elucidate the impact of RNSP on hypoxia-induced neuropathology, we examined key molecular and histological markers in the brains of C57BL/6J mice following a 28-day chronic hypoxia exposure, with or without a 7-day RNSP pretreatment. Molecular analysis of hippocampal and cortical lysates by RT-qPCR demonstrated that chronic hypoxia significantly elevated the mRNA levels of pro-inflammatory cytokines (IL-1β, IL-6, and TNF-α) and the amyloid precursor protein (APP), compared to normoxic controls (**Figure 2**). RNSP treatment potently reversed these molecular alterations, significantly downregulating the expression of these inflammatory mediators and APP at both the transcriptional and translational levels. Corroborating these findings, histological examination of brain sections via H&E staining revealed distinct pathological changes in specific regions—including the CA1, CA3, and DG subfields of the hippocampus and the primary somatosensory cortex (S1)—in hypoxic mice, which were consistent with the pattern of elevated APP and cytokine expression (**Figure 3**). Notably, these histopathological aberrations were markedly attenuated in the RNSP-treated group, presenting a cellular architecture that more closely resembled the normoxic controls. Collectively, these integrated molecular and histological data provide compelling evidence that RNSP effectively alleviates chronic hypoxia-driven neuroinflammation and amyloid-beta pathology in a region-specific manner within the mouse brain.

### 3.3 RNSP suppresses systemic inflammation in peripheral tissues

**Figure 4:**
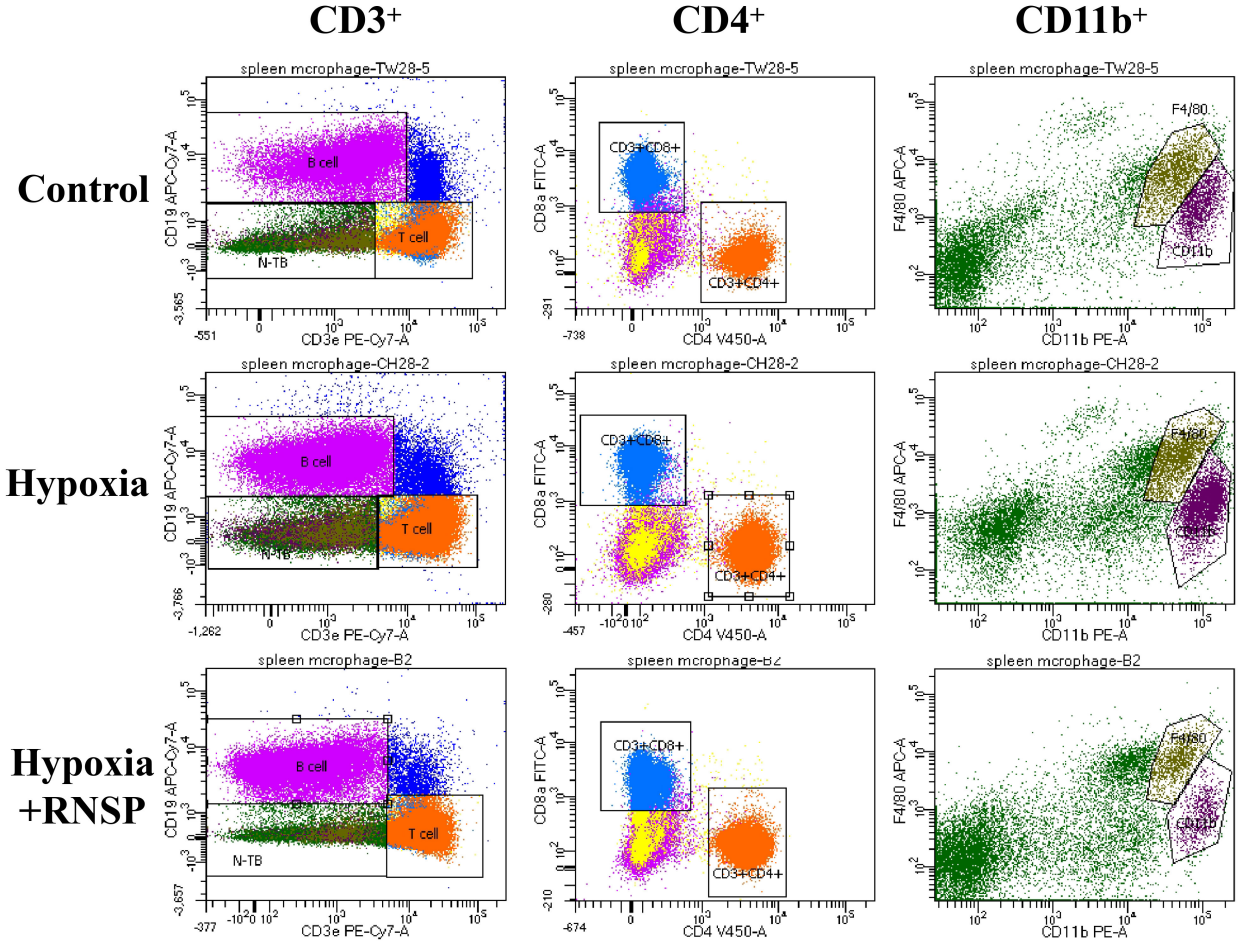
RNSP regulated the chronic hypoxia-increased T cells and macrophages accumulation in spleen. Flow cytometry was used to detect the levels of CD3^+^, CD4^+^, and CD11b^+^ in mouse spleen tissue. CD3^+^ was labeled with BD pharmingen™ PE-Cy™ 7 Hamster Anti-Mouse, CD4^+^ with BD pharmingen™ Pacific Blue™ Rat Anti-Mouse, and CD11b^+^ with BD pharmingen™ PE Rat Anti-Mouse. Compared with the control group, the expression of CD3^+^ and CD4^+^ in the spleen tissue of mice in the hypoxia group was significantly lower, while the expression of CD11b^+^ was increased. After 28 days of RNSP treatment, the expression of CD3^+^ and CD4^+^ was significantly increased, while the expression of CD11b^+^ was significantly decreased.

**Figure 5:**
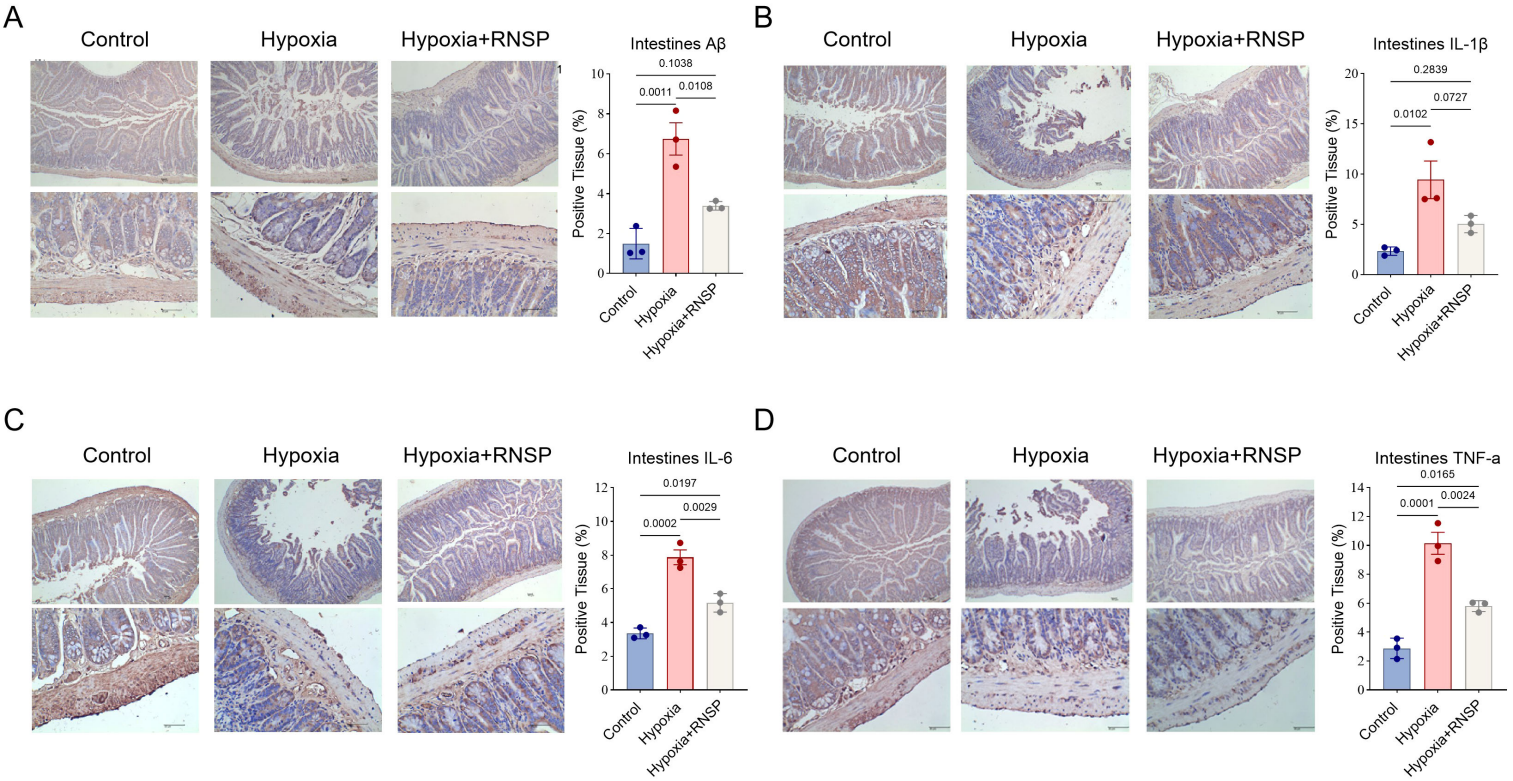
RNSP suppressed the chronic hypoxia-increased inflammation in gut. **(A-D)** Immunohistochemical staining was used to observe the expression of Aβ, IL-1β, IL-6, and TNF-α in the intestine tissue of mice. Compared with the control group, the expression of Aβ, IL-1β, IL-6, and TNF-α in the hypoxia group was increased. After 28 days of RNSP treatment, the expression of Aβ, IL-1β, IL-6, and TNF-α was significantly reduced. Data are presented as mean ± SD. Statistical significance was determined by one-way ANOVA followed by an appropriate *post-hoc* test, with p < 0.05 considered significant. Exact p-values are shown directly above the bars.

The anti-inflammatory effects of RNSP were further evaluated in peripheral tissues of C57BL/6J mice subjected to 28-day chronic hypoxia, following a 7-day RNSP pretreatment. In the spleen, RT-qPCR analysis revealed that chronic hypoxia significantly upregulated the mRNA expression of pro-inflammatory cytokines (IL-1β, IL-6, and TNF-α) and the amyloid precursor protein (APP), RNSP treatment effectively normalized these transcript levels (**Table 1**). Consistent with this, flow cytometric analysis of splenic tissue demonstrated that hypoxia-induced reduction in the abundance of T cells (identified by CD3^+^ and CD4^+^) and myeloid cells (identified by CD11b^+^) were markedly reversed by RNSP administration (**Figure 4**). Furthermore, histopathological examination of the intestine by immunohistochemistry revealed a pronounced increase in the immunoreactivity of Aβ, IL-1β, IL-6, and TNF-α in the hypoxic group compared to controls (**Figure 5**). This peripheral inflammatory response in the gut was substantially mitigated in RNSP-treated mice, which exhibited staining intensities for these markers comparable to baseline levels. Collectively, these data from the spleen and gut demonstrate that RNSP robustly suppresses chronic hypoxia-induced systemic inflammation by modulating immune cell populations and reducing the expression of key inflammatory mediators and APP/Aβ in peripheral tissues.

**Table 1:** The mRNA expression of APP, IL-1β, IL-6, and TNF-α in mouse spleen tissue.

Groups	n	Relative mRNA expression			
		APP	IL-1β	IL-6	TNF-a
Control	3	0.893 ± 0.176	0.980 ± 0.070	0.797 ± 0.234	1.030 ± 0.305
Hypoxia	3	2.250 ± 0.330**	1.963 ± 0.511*	1.677 ± 0.363**	2.640 ± 0.214**
Hypoxia+RNSP	3	1.883 ± 0.628*	1.503 ± 0.210	1.387 ± 0.189*	2.207 ± 0.712*
F value		8.294	7.038	8.149	9.663
P value		0.019	0.027	0.019	0.013

*p < 0.05, **p < 0.01 vs. Control group.

### 3.4 RNSP modulates immune cell populations and activation in the periphery

**Figure 6:**
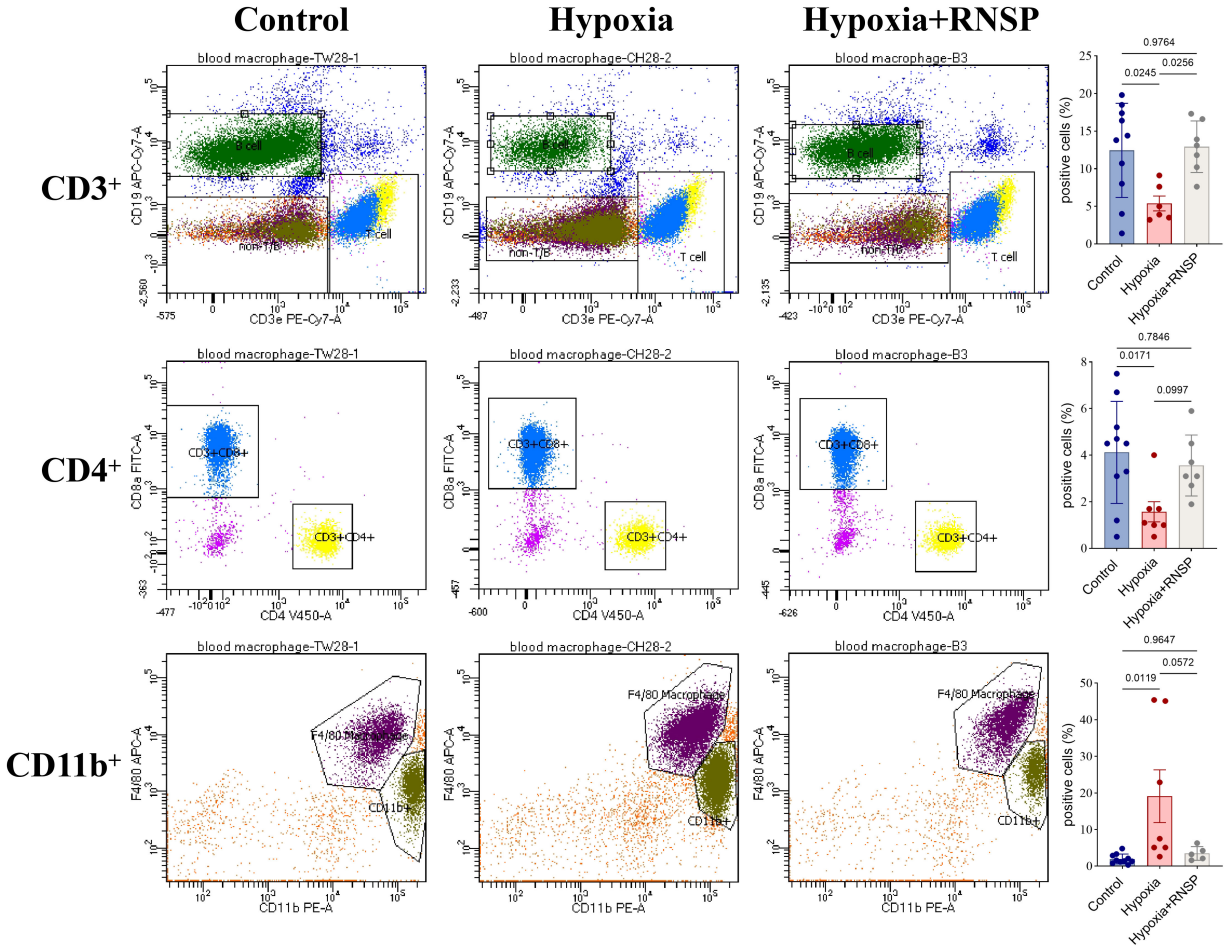
RNSP regulated the chronic hypoxia-increased T cells and macrophages accumulation in peripheral blood. Flow cytometry was used to detect the levels of CD3^+^, CD4^+^, and CD11b^+^ in serum of mice. BD pharmingen™ PE-Cy™ 7 Hamster Anti-Mouse was used to label CD3^+^, BD pharmingen™ Pacific Blue™ Rat Anti-Mouse to label CD4^+^, and BD pharmingen™ PE Rat Anti-Mouse to label CD11b^+^. Compared with the control group, the expression of CD3^+^ and CD4^+^ in serum in the hypoxia group was significantly decreased, while the expression of CD11b^+^ was increased. After 28 days of RNSP treatment, the expression of CD3^+^ and CD4^+^ was significantly increased, while the expression of CD11b^+^ showed no statistically significant difference. Data are presented as mean ± SD. Statistical significance was determined by one-way ANOVA followed by an appropriate *post-hoc* test, with p < 0.05 considered significant. Exact p-values are shown directly above the bars.

To systemically assess the immunomodulatory effect of RNSP, we analyzed serum cytokine profiles and immune cell populations in the peripheral blood of C57BL/6J mice following a 28-day chronic hypoxia paradigm with or without a 7-day RNSP pretreatment. ELISA analysis of serum revealed a pronounced systemic inflammatory state in hypoxic mice, characterized by significantly elevated levels of pro-inflammatory mediators, including IL-1β, IL-6, and TNF-α. Concurrently, the levels of anti-inflammatory cytokines such as IL-4, IL-10, and TGF-β were markedly reduced. Serum Aβ levels did not differ significantly among groups (**Table 2**). RNSP treatment effectively counteracted this immunological imbalance, significantly suppressing the hypoxia-induced pro-inflammatory cytokine surge and restoring the circulating levels of anti-inflammatory cytokines. Corroborating these findings, flow cytometric analysis of peripheral blood showed that chronic hypoxia led to a significant decrease in the frequencies of total T cells (CD3^+^) and helper T cells (CD4^+^), while increase myeloid cells (CD11b^+^) (**Figure 6**). This expansion of key immune cell populations was notably reversed by RNSP administration. Collectively, these results demonstrate that RNSP ameliorates chronic hypoxia-induced systemic inflammation by rebalancing the pro- and anti-inflammatory cytokine milieu and normalizing the distribution of peripheral immune cells.

**Table 2:** RNSP suppressed the chronic hypoxia-increased inflammation in serum.

Item (pg/ml)	Control(n=3)	Hypoxic(n=3)	Hypoxic+RNSP(n=3)	F value	P value
Aβ	175.622 ± 8.937	185.370 ± 7.601	183.649 ± 9.544	1.065	0.402
IL-1β	26.311 ± 2.930	33.263 ± 1.713**	28.323 ± 2.026#	7.374	0.024
IL-6	44.435 ± 3.348	52.921 ± 2.165*	47.222 ± 2.002#	8.459	0.018
TNF-a	230.187 ± 36.534	299.904 ± 6.961**	245.045 ± 13.267#	7.802	0.021
IL-4	32.775 ± 2.342	25.464 ± 0.733**	29.511 ± 1.145*#	16.462	0.004
IL-10	50.739 ± 6.686	31.186 ± 1.074**	38.578 ± 1.034**	18.695	0.003
IL-17	8.450 ± 0.775	5.552 ± 0.830**	6.964 ± 0.259*#	13.934	0.006
TGF-β	5.088 ± 0.529	2.624 ± 0.220**	4.042 ± 0.369*##	28.462	0.001

*p < 0.05, **p < 0.01 vs. Control group; #p < 0.05, ##p < 0.01 vs. Hypoxia group.

## 4 Discussion

**Figure 7:**
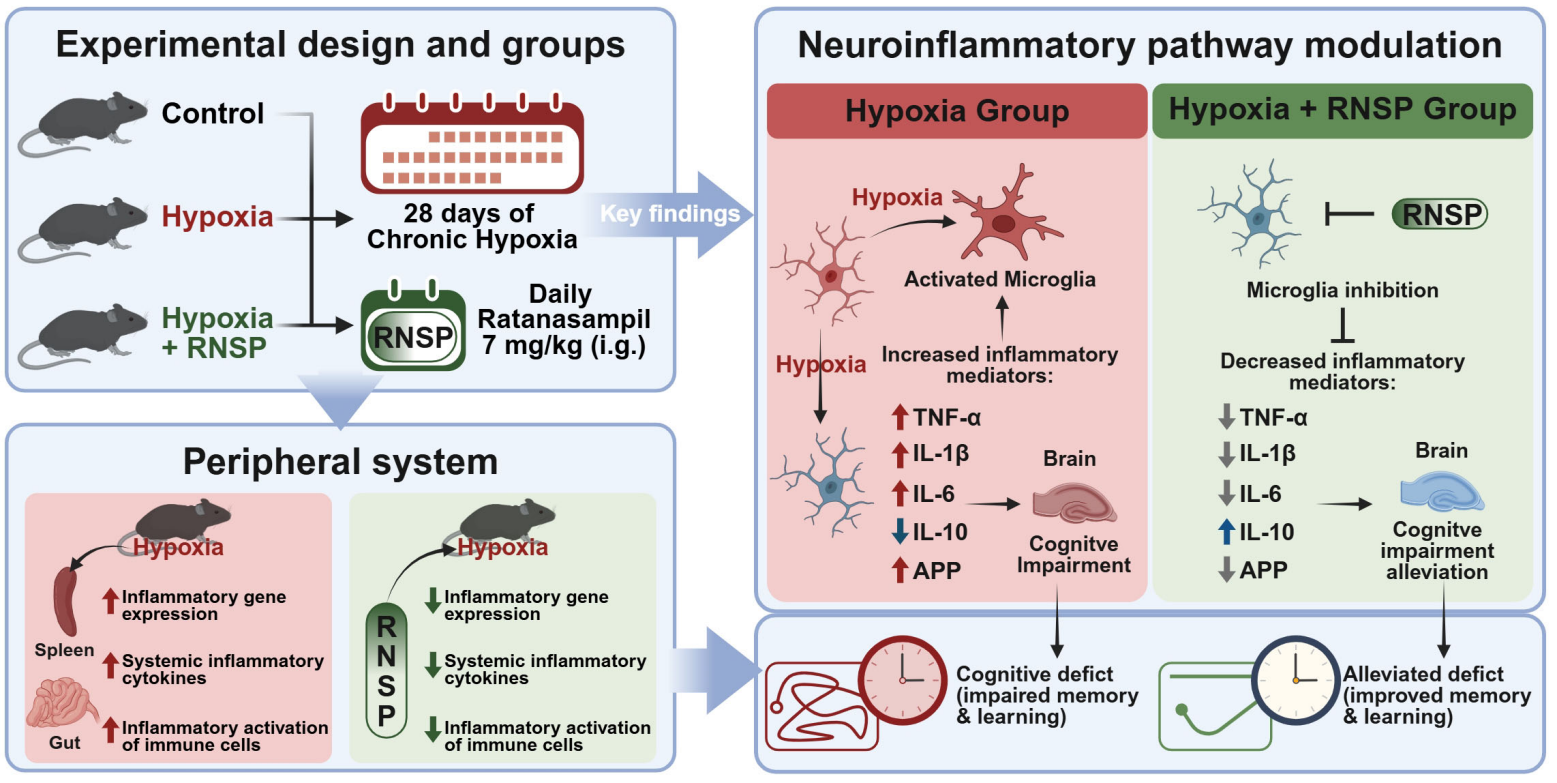
Schematic diagram of illustrating the protective effects of RNSP against chronic hypoxia-induced cognitive impairment. Chronic hypoxia initiates peripheral inflammation by inducing gut dysbiosis and splenic immune activation, which elevates systemic pro-inflammatory cytokines that cross the blood-brain barrier to drive neuroinflammation. Within the hippocampus and cortex, this cascade leads to microglial overactivation, amyloid-beta (Aβ) accumulation, and increased levels of pro-inflammatory mediators (TNF-α, IL-1β, IL-6), resulting in neuronal damage and cognitive decline. RNSP administration (7 mg/kg/d) exerts dual anti-inflammatory actions by mitigating systemic inflammatory inputs from the gut and spleen and inhibiting hippocampal neuroinflammatory pathways, thereby reducing Aβ pathology, rebalancing the cytokines, and providing neuroprotection.

In this study, we have demonstrated that RNSP effectively mitigates the cognitive deficits caused by chronic hypoxia. Beyond its role in improving cognition, RNSP significantly attenuated neuroinflammation and amyloid beta accumulation in the cortex and hippocampus. Additionally, RNSP exhibited anti-inflammatory effects in peripheral tissues, including the spleen, gut, and immune cells such as T cells and macrophages (**Figure 7**). These results highlight the dual central and peripheral anti-inflammatory actions of RNSP, supporting its potential as a therapeutic agent for addressing cognitive impairments and systemic inflammation associated with chronic hypoxia, especially in high-altitude environments.

One of the key findings of this study is the significant reduction in hypoxia-induced neuroinflammation by RNSP. However, whether RNSP can cross the blood-brain barrier (BBB) and directly act within the central nervous system remains unclear. The observed reduction in neuroinflammation may arise either from direct actions on brain-resident cells or indirectly through its suppression of systemic inflammation. Previous studies have demonstrated that neuroinflammation in chronic hypoxia is mediated by activated microglia and astrocytes, which release pro-inflammatory cytokines and exacerbate neuronal injury (24, 25). In this context, Tibetan herbal formulations, including compounds like RNSP, have shown potential to reduce neuroinflammation in other neurological conditions, although the precise mechanisms underlying these effects remain poorly understood (21, 23). Previous work has shown that hypoxia exacerbates the accumulation of β-amyloid in the brain, a process that contributes to cognitive decline and neuroinflammation (26, 27). RNSP’s ability to suppress this accumulation of β-amyloid adds an important layer to its neuroprotective effects, potentially modulating amyloid metabolism via inflammatory pathways or direct cellular interactions. If RNSP cannot cross the BBB, its anti-inflammatory effects on peripheral immune cells, such as T cells and macrophages, may help modulate brain inflammation indirectly through systemic immune signaling (28). Future studies using pharmacokinetic analysis and BBB permeability assays are needed to determine whether RNSP acts directly on neural tissue or through peripheral mechanisms. Understanding this distinction is crucial for developing RNSP as a therapeutic strategy for high-altitude-induced cognitive decline.

Our data demonstrate that chronic hypoxia leads to a significant reduction in CD3^+^ and CD4^+^ T cells in both spleen and peripheral blood, accompanied by elevated CD11b^+^ myeloid cells, indicating systemic immune activation. RNSP treatment normalized these populations, suggesting an immunomodulatory effect on both adaptive and innate immunity. These findings align with previous reports that chronic hypoxia can induce splenic T cell expansion and myeloid cell recruitment, which are thought to contribute to the propagation of peripheral inflammation to the CNS (29, 30). Interestingly, while CD11b^+^ cells were reduced in the spleen by RNSP, the reduction in peripheral blood did not reach statistical significance (**Figure 6**), implying that RNSP may exert tissue-specific effects or that the timing of analysis may have captured only partial recovery. The mechanism by which RNSP modulates T cell and macrophage activation warrants further investigation. One possibility is through the inhibition of NF−κB and MAPK signaling pathways, as has been shown for other Tibetan medicinal herbs (31).

Our study expands on this body of work by exploring the potential of RNSP in the context of hypoxia-induced inflammatory diseases. Chronic hypoxia, a condition that exacerbates neuroinflammation, particularly in the brain, has been implicated in various diseases, including AD and ischemic stroke. The gut represents another critical peripheral site where RNSP exerts anti-inflammatory activity. Chronic hypoxia is known to compromise intestinal barrier function by reducing tight junction protein expression, leading to increased translocation of lipopolysaccharide (LPS) and subsequent systemic endotoxemia (32). Our immunohistochemical results showed that RNSP markedly decreased the expression of Aβ, IL−1β, IL−6, and TNF−α in the gut wall (**Figure 5**), suggesting that RNSP protects against hypoxia−induced gut inflammation. This finding is particularly important because gut−derived inflammatory mediators can access the brain via the vagus nerve or the systemic circulation, thereby perpetuating neuroinflammation (18, 19). Thus, RNSP may interrupt the gut−brain inflammatory axis, providing an additional mechanism for its cognitive benefits. Future studies should directly measure intestinal permeability and assess gut microbiota composition to determine whether RNSP modulates the microbiome.

The serum cytokine profile revealed a striking imbalance between pro-and anti−inflammatory mediators after chronic hypoxia (**Table 2**). RNSP significantly lowered the elevated levels of IL−1β, IL−6, and TNF−α, while restoring the depleted levels of IL−4, IL−10, and TGF−β. This rebalancing effect is reminiscent of the action of some natural products that act as “multitarget” immunomodulators (33). Notably, chronic hypoxia significantly reduced serum IL-17 levels, and RNSP treatment partially restored them (**Table 2**). Although IL-17 is generally considered a pro-inflammatory cytokine capable of disrupting the blood-brain barrier and activating microglia (34, 35), the functional implications of its reduction under hypoxic conditions remain unclear. It is possible that hypoxia-induced immune dysregulation leads to aberrant suppression of Th17 responses, and RNSP may help restore immune homeostasis. Whether RNSP directly affects Th17 cell differentiation or indirectly modulates the cytokine milieu through antigen-presenting cells requires further investigation.

We propose that RNSP could serve as a promising therapeutic agent in these conditions by modulating the immune response and reducing inflammation in both the brain and peripheral tissues. Our findings suggest that RNSP treatment effectively suppresses hypoxia-induced inflammatory responses, both in the central nervous system and in peripheral tissues, such as the spleen and gut. This adds a novel dimension to the therapeutic potential of RNSP in treating hypoxia-induced inflammatory diseases, emphasizing its broader applicability in clinical settings involving chronic inflammatory states. In summary, this study demonstrates that RNSP ameliorates chronic hypoxia-induced cognitive impairment in mice by suppressing neuroinflammation and reducing amyloid-β accumulation in the brain, while simultaneously dampening systemic inflammation in the spleen, gut, and peripheral blood. The dual anti-inflammatory action of RNSP, targeting both central and peripheral compartments, positions it as a promising candidate for managing cognitive decline in high-altitude environments.

## Data Availability

The raw data supporting the conclusions of this article will be made available by the authors, without undue reservation.
